# Supplementary material for: Disruption of the CRF1 receptor eliminates morphine-induced sociability deficits and firing of oxytocinergic neurons in male mice
Source: eLife. 2025 Feb 5;13:RP100849. doi: 10.7554/eLife.100849 (PMC11798570; doi:10.7554/eLife.100849)
Supplement: Supplementary file 1. — (a–c) Number of animals used and cells patched and recorded. (d) Statistical analysis of the three-chamber sociability test in C57BL/6J mice. (e) Statistical analysis of locomotor activity displayed by C57BL/6J mice during the three-chamber sociability test. (f) Statistical analysis of the three-chamber sociability test in CRF1 receptor-deficient mice. (g) Female CRF1 WT and CRF1 HET mice fail to perform in the three-chamber task for sociability. (h) Statistical analysis of neuronal firing in C57BL/6J mice. [file elife-100849-supp1.docx]

**Supplementary File 1**

Manuscript title:

**Disruption of the CRF_1_ receptor eliminates morphine-induced sociability deficits and firing of oxytocinergic neurons in male mice**

Authors:

**Alessandro Piccin^1,2^, Anne-Emilie Allain^1,2^, Jérôme Baufreton^4,5^, Sandrine S. Bertrand^1,2^, Angelo Contarino^1,2,3^**

^1^ Université de Bordeaux, INCIA, UMR 5287, 33076 Bordeaux, France

^2^ CNRS, INCIA, UMR 5287, 33076 Bordeaux, France

^3^ INSERM, T3S, UMR-S 1124, Université Paris Cité, 75006 Paris, France

^4^ Université de Bordeaux, IMN, UMR 5293, 33076 Bordeaux, France

^5^ CNRS, IMN, UMR 5293, 33076 Bordeaux, France

List of the supplementary files:

**Supplementary files 1a-c. Number of animals used and cells patched and recorded**.

**Supplementary file 1d. Statistical analysis of the three-chamber sociability test in C57BL/6J mice**.

**Supplementary file 1e. Statistical analysis of locomotor activity displayed by C57BL/6J mice during the three-chamber sociability test**.

**Supplementary file 1f. Statistical analysis of the three-chamber sociability test in CRF_1_ receptor-deficient mice**.

**Supplementary file 1g. Female CRF_1_ WT and CRF_1_ HET mice fail to perform in the three-chamber task for sociability**.

**Supplementary file 1h. Statistical analysis of neuronal firing in C57BL/6J mice**.

**Supplementary files 1a-c. Number of animals used and cells patched and recorded**. **a)** Number of male and female C57BL/6J mice tested in the three-chamber task after being treated *per os* (p.o.) with either vehicle (veh) or antalarmin (anta; 20 mg/kg) and intraperitoneally (i.p.) with either saline (sal) or morphine (mor; 2.5 mg/kg). **b)** Number of saline- or morphine (0.625 mg/kg)-treated male CRF_1_ WT, CRF_1_ HET and CRF_1_ KO mice tested in the three-chamber task and number of cells patched and recorded in the electrophysiology studies. In brackets, the number of animals excluded from the statistical analysis of the data because met the exclusion criterion, i.e., exploration of each region of interest (ROI, side half-chamber) of the three-chamber apparatus for more than 80%, or less than 20%, of the total time spent in both ROIs during the habituation phase. **c)** Number of patched and recorded paraventricular nucleus of the hypothalamus (PVN) neurons expressing oxytocin (OXY) and/or arginine-vasopressin (AVP) or neither OXY nor AVP (no staining) in male (M) and female (F) C57BL/6J mice treated with either vehicle or antalarmin followed by either saline or morphine, as in **a)**.

**a)**

| **C57BL/6J** | **Veh/sal** | **Anta/sal** | **Veh/mor** | **Anta/mor** |
| --- | --- | --- | --- | --- |
| **Male** | 9 (0) | 9 (1) | 12 (3) | 12 (2) |
| **Female** | 7 (1) | 8 (1) | 7 (1) | 9 (3) |

**b)**

|  | **Three-chamber** | | **Electrophysiology** | |
| --- | --- | --- | --- | --- |
| **Genotype** | **Sal** | **Mor** | **Sal** | **Mor** |
| **CRF_1_ WT** | 12 (3) | 11 (2) | 18 | 21 |
| **CRF_1_ HET** | 16 (3) | 17 (6) | 21 | 31 |
| **CRF_1_ KO** | 13 (1) | 12 (2) | 17 | 17 |

**c)**

|  | **Veh/sal** | | **Anta/sal** | | **Veh/mor** | | **Anta/mor** | | |
| --- | --- | --- | --- | --- | --- | --- | --- | --- | --- |
| **C57BL/6J** | **M** | **F** | **M** | **F** | **M** | **F** | | **M** | **F** |
| **OXY/AVP** | 7 | 7 | 8 | 7 | 11 | 8 | | 14 | 9 |
| **AVP** | 13 | 2 | 16 | 2 | 12 | 2 | | 8 | 1 |
| **OXY** | 2 | 11 | 1 | 7 | 1 | 8 | | 2 | 12 |
| **No staining** | 2 | 6 | 4 | 4 | 4 | 4 | | 5 | 3 |
| ***Total* cells** | **24** | **26** | **29** | **20** | **28** | **22** | | **29** | **25** |

**Supplementary file 1d. Statistical analysis of the three-chamber sociability test in C57BL/6J mice**. Statistical analysis of time (s) spent in the regions of interest (ROIs, side half-chambers) of the three-chamber apparatus by male and female C57BL/6J mice treated *per os* (p.o.) with either vehicle or antalarmin (20 mg/kg) and intraperitoneally (i.p.) with either saline or morphine (2.5 mg/kg), during the habituation or the sociability phase of the three-chamber test. Pretreatment (P): vehicle *vs*. antalarmin. Treatment (T): saline *vs*. morphine. Repeated measures (RM): mouse *vs.* object. Further details are reported in the “statistical analysis” section of the manuscript.

|  | **Male** | | **Female** | |
| --- | --- | --- | --- | --- |
|  | **Habituation** | **Sociability** | **Habituation** | **Sociability** |
| **P** | F_1,32_=1.122  P=0.297 | F_1,32_=4.075  P=0.052 | F_1,21_=1.692  P=0.207 | F_1,21_=1.296  P=0.268 |
| **T** | F_1,32_=0.064  P=0.802 | F_1,32_=0.438  P=0.513 | F_1,21_=23.501  P<0.0001 | F_1,21_=25.077  P<0.0001 |
| **P x T** | F_1,32_=1.025  P=0.319 | F_1,32_=11.590  P<0.005 | F_1,21_=0.253  P=0.620 | F_1,21_=0.290  P=0.596 |
| **RM** | F_1,32_=0.001  P=0.976 | F_1,32_=23.795  P<0.0001 | F_1,21_=0.005  P=0.942 | F_1,21_=4.547  P<0.05 |
| **P x RM** | F_1,32_=0.128  P=0.723 | F_1,32_=8.977  P<0.01 | F_1,21_=0.024  P=0.879 | F_1,21_=0.032  P=0.860 |
| **T x RM** | F_1,32_=0.240  P=0.628 | F_1,32_=1.225  P=0.277 | F_1,21_=0.010  P=0.922 | F_1,21_=14.218  P<0.005 |
| **P x T x RM** | F_1,32_=0.163  P=0.689 | F_1,32_=8.444  P<0.01 | F_1,21_=0.002  P=0.965 | F_1,21_=0.037  P=0.848 |

**Supplementary file 1e. Statistical analysis of locomotor activity displayed by C57BL/6J mice during the three-chamber sociability test**. Statistical analysis of distance (m) travelled by male and female C57BL/6J mice treated *per os* (p.o.) with either vehicle or antalarmin (20 mg/kg) and intraperitoneally (i.p.) with either saline or morphine (2.5 mg/kg), during the habituation and the sociability phases of the three-chamber test. Pretreatment (P): vehicle *vs*. antalarmin. Treatment (T): saline *vs*. morphine. Repeated measures (RM): habituation *vs.* sociability phase. Further details are reported in the “statistical analysis” section of the manuscript.

|  | **Male** | **Female** |
| --- | --- | --- |
| **P** | F_1,32_=0.799  P=0.378 | F_1,21_=0.107  P=0.746 |
| **T** | F_1,32_=6.648  P<0.05 | F_1,21_=1.485  P=0.237 |
| **P x T** | F_1,32_=0.401  P=0.531 | F_1,21_=0.159  P=0.694 |
| **RM** | F_1,32_=9.779  P<0.005 | F_1,21_=6.055  P<0.05 |
| **P x RM** | F_1,32_=1.708  P=0.201 | F_1,21_=0.431  P=0.519 |
| **T x RM** | F_1,32_=1.431  P=0.240 | F_1,21_=3.929  P=0.061 |
| **P x T x RM** | F_1,32_=0.482  P=0.492 | F_1,21_=0.143  P=0.709 |

**Supplementary file 1f. Statistical analysis of the three-chamber sociability test in CRF_1_ receptor-deficient mice**. Statistical analysis of time (s) spent in the regions of interest (ROIs, side half-chambers) of the three-chamber apparatus and distance (m) travelled during the habituation and the sociability phases of the three-chamber test by male CRF_1_ WT, CRF_1_ HET and CRF_1_ KO mice treated intraperitoneally (i.p.) with either saline or morphine (0.625 mg/kg). Genotype (G): CRF_1_ WT *vs*. CRF_1_ HET *vs*. CRF_1_ KO. Treatment (T): saline *vs*. morphine. Repeated measures (RM): mouse *vs.* object for the habituation and the sociability phase, habituation *vs.* sociability phase for distance. Further details are reported in the “statistical analysis” section of the manuscript.

|  | **Habituation** | **Sociability** | **Distance** |
| --- | --- | --- | --- |
| **G** | F_2,58_=2.528  P=0.089 | F_2,58_=1.464  P=0.240 | F_2,58_=2.280  P=0.111 |
| **T** | F_1,58_=2.440  P=0.124 | F_1,58_=0.002  P=0.963 | F_1,58_=0.942  P=0.336 |
| **G x T** | F_2,58_=5.069  P<0.01 | F_2,58_=1.473  P=0.238 | F_2,58_=1.525  P=0.226 |
| **RM** | F_1,58_=0.023  P=0.879 | F_1,58_=48.492  P<0.0001 | F_1,58_=82.681  P<0.0001 |
| **G x RM** | F_2,58_=0.019  P=0.981 | F_2,58_=2.278  P=0.112 | F_2,58_=1.772  P=0.179 |
| **T x RM** | F_1,58_=0.031  P=0.861 | F_1,58_=7.504  P<0.01 | F_1,58_=2.000  P=0.163 |
| **G x T x RM** | F_2,58_=0.083  P=0.920 | F_2,58_=5.261  P<0.01 | F_2,58_=5.145  P<0.01 |

**Supplementary file 1g. Female CRF_1_ WT and CRF_1_ HET mice fail to perform in the three-chamber task for sociability.** Number of female CRF_1_ WT and CRF_1_ HET mice treated intraperitoneally (i.p.) with either saline or morphine (0.625 mg/kg) that visited both, only one or neither of the two side chambers of the three-chamber apparatus during the habituation phase (10 min) of the three-chamber test. Notably, only 2/8 saline-treated CRF_1_ WT, 2/8 morphine-treated CRF_1_ WT and 3/8 morphine-treated CRF_1_ HET female mice visited both side chambers of the apparatus. Thus, most of the animals tested met the exclusion criterion, i.e., exploration of each region of interest (ROI, side half-chamber) of the three-chamber apparatus for more than 80%, or less than 20%, of the total time spent in both ROIs during the habituation phase. This made impossible to assess morphine effects upon social behavior in female CRF_1_ receptor-deficient mice using a reasonable number of animals.

|  | **Side chambers visited** | | |
| --- | --- | --- | --- |
| **Genotype and treatment** | **Both** | **One** | **Neither** |
| **CRF_1_ WT saline (n=8)** | 2 | 2 | 4 |
| **CRF_1_ WT morphine (n=8)** | 2 | 3 | 3 |
|  |  |  |  |
| **CRF_1_ HET saline (n=4)** | 4 | 0 | 0 |
| **CRF_1_ HET morphine (n=8)** | 3 | 3 | 2 |

**Supplementary file 1h. Statistical analysis of neuronal firing in C57BL/6J mice**. Statistical analysis of firing frequency (Hz) displayed by paraventricular nucleus of the hypothalamus (PVN) neurons expressing oxytocin (OXY) and/or arginine-vasopressin (AVP) in male and female C57BL/6J mice treated *per os* (p.o.) with either vehicle or antalarmin (20 mg/kg) and intraperitoneally (i.p.) with either saline or morphine (2.5 mg/kg). Pretreatment (P): vehicle *vs*. antalarmin. Treatment (T): saline *vs*. morphine. Further details are reported in the “statistical analysis” section of the manuscript.

|  | **Male** | | **Female** | |
| --- | --- | --- | --- | --- |
|  | **OXY/AVP** | **AVP** | **OXY/AVP** | **OXY** |
| **P** | F_1,36_=10.201  P<0.005 | F_1,45_=0.340  P=0.562 | F_1,27_=0.465  P=0.501 | F_1,34_=0.094  p=0.761 |
| **T** | F_1,36_=17.133  P<0.0005 | F_1,45_=5.173  P<0.05 | F_1,27_=13.685  P<0.001 | F_1,34_=10.031  P<0.005 |
| **P x T** | F_1,36_=10.186  P<0.005 | F_1,45_=0.804  P=0.375 | F_1,27_=0.006  P=0.939 | F_1,34_=1.145  P=0.292 |
