# Supplementary material for: Disruption of the CRF1 receptor eliminates morphine-induced sociability deficits and firing of oxytocinergic neurons in male mice
Source: eLife. 2025 Feb 5;13:RP100849. doi: 10.7554/eLife.100849 (PMC11798570; doi:10.7554/eLife.100849)
Supplement: MDAR checklist [file elife-100849-mdarchecklist1.docx]

**Materials Design Analysis Reporting (MDAR)**

**Checklist for Authors**

The [MDAR framework](https://osf.io/xfpn4/) establishes a minimum set of requirements in transparent reporting mainly applicable to studies in the life sciences.

*eLife* asks authors to **provide detailed information within their article** to facilitate the interpretation and replication of their work. Authors can also upload supporting materials to comply with relevant reporting guidelines for health-related research (see [EQUATOR Network](http://www.equator-network.org/%20)), life science research (see the [BioSharing Information Resource](http://biosharing.org/)), or animal research (see the [ARRIVE Guidelines](http://www.plosbiology.org/article/info:doi/10.1371/journal.pbio.1000412) and the [STRANGE Framework](https://doi.org/10.1038/d41586-020-01751-5); for details, see *eLife*’s [Journal Policies](https://reviewer.elifesciences.org/author-guide/journal-policies)). Where applicable, authors should refer to any relevant reporting standards materials in this form.

For all that apply, please note **where in the article** the information is provided. Please note that we also collect information about data availability and ethics in the submission form.

**Materials:**

| **Newly created materials** | **Indicate where provided: section/figure legend** | **N/A** |
| --- | --- | --- |
| The manuscript includes a dedicated "materials availability statement" providing transparent disclosure about availability of newly created materials including details on how materials can be accessed and describing any restrictions on access. |  | X |
|  |  |  |
| **Antibodies** | **Indicate where provided: section/figure legend** | **N/A** |
| For commercial reagents, provide supplier name, catalogue number and [RRID](https://scicrunch.org/resources), if available.  Mouse anti-OXY monoclonal antibody (1/1000, Millipore MAB5296)  T-5048 Guinea pig anti (Arg^8^)-vasopressin antibody (1/1000, BMA biomedicals)  Alexa fluor 488 goat anti-mouse IgG (1/500, Life technology)  Alexa fluor 647 donkey anti-guinea pig IgG (1/500, Life technology) | Materials and methods (*Immunohistochemistry and imaging*) |  |
|  |  |  |
| **DNA and RNA sequences** | **Indicate where provided: section/figure legend** | **N/A** |
| Short novel DNA or RNA including primers, probes: Sequences should be included or deposited in a public repository. |  | X |
|  |  |  |
| **Cell materials** | **Indicate where provided: section/figure legend** | **N/A** |
| Cell lines: Provide species information, strain. Provide accession number in repository OR supplier name, catalog number, clone number, OR RRID. |  | X |
| Primary cultures: Provide species, strain, sex of origin, genetic modification status. |  | X |
|  |  |  |
| **Experimental animals** | **Indicate where provided: section/figure legend** | **N/A** |
| Laboratory animals or Model organisms: Provide species, strain, sex, age, genetic modification status. Provide accession number in repository OR supplier name, catalog number, clone number, OR RRID.  Mouse; C57BL/6J; males and females; 12-28 weeks old at testing, CRF1 receptor KO | Materials and methods (*Animals*) |  |
| Animal observed in or captured from the field: Provide species, sex, and age where possible. |  | X |
|  |  |  |
| **Plants and microbes** | **Indicate where provided: section/figure legend** | **N/A** |
| Plants: provide species and strain, ecotype and cultivar where relevant, unique accession number if available, and source (including location for collected wild specimens). |  | X |
| Microbes: provide species and strain, unique accession number if available, and source. |  | X |
|  |  |  |
| **Human research participants** | **Indicate where provided: section/figure legend) or state if these demographics were not collected** | **N/A** |
| If collected and within the bounds of privacy constraints report on age, sex, gender and ethnicity for all study participants. |  | X |

**Design:**

| **Study protocol** | **Indicate where provided: section/figure legend** | **N/A** |
| --- | --- | --- |
| If the study protocol has been pre-registered, provide DOI. For clinical trials, provide the trial registration number OR cite DOI. |  | X |
|  |  |  |
| **Laboratory protocol** | **Indicate where provided: section/figure legend** | **N/A** |
| Provide DOI OR other citation details if detailed step-by-step protocols are available. |  | X |
|  |  |  |
| **Experimental study design (statistics details) *** | | |
| **For in vivo studies: State whether and how the following have been done** | **Indicate where provided: section/figure legend. If it could have been done, but was not, write “not done”** | **N/A** |
| Sample size determination  Sample sizes were determined based on prior studies from our laboratory using the same techniques. | Not mentioned in the manuscript. |  |
| Randomisation  The three-chamber experiments were generally carried out over a 5-day period. In particular, on each test day approximately the same number of mice was assigned to each experimental group. | Materials and methods (*Three-chamber sociability task*) |  |
| Blinding  Each mouse was assigned a unique identification number that was used to conduct blind testing and data analysis. | Materials and methods (*Statistical analysis*) |  |
| Inclusion/exclusion criteria  To prevent strong initial preferences from biasing the three-chamber sociability results, animals exploring each region of interest (ROI) containing the wire cage for more than 80% (or less than 20%) of the total time spent in both ROIs during the habituation phase (10 min) were excluded from data analysis. The number of animals excluded within each experimental group is reported in the **Supplementary files 1a-b**. | Materials and methods (*Statistical analysis*) |  |
|  |  |  |
| **Sample definition and in-laboratory replication** | **Indicate where provided: section/figure legend** | **N/A** |
| State number of times the experiment was replicated in the laboratory.  Experiments were replicated 2-3 times. Given the similar results obtained across the replicates, data were finally pooled up. | Not mentioned in the manuscript. |  |
| Define whether data describe technical or biological replicates.  The data obtained are provided.  Biological replicates. |  |  |
|  |  |  |
| **Ethics** | **Indicate where provided: section/submission form** | **N/A** |
| Studies involving human participants: State details of authority granting ethics approval (IRB or equivalent committee(s), provide reference number for approval. |  | X |
| Studies involving experimental animals: State details of authority granting ethics approval (IRB or equivalent committee(s), provide reference number for approval.  All studies were conducted in accordance with the European Communities Council Directive 2010/63/EU, were approved by the local Animal Care and Use Committee of the University of Bordeaux (DAP13274) and complied with the ARRIVE Guidelines. | Materials and methods (*Animals*) |  |
| Studies involving specimen and field samples: State if relevant permits obtained, provide details of authority approving study; if none were required, explain why. |  | X |
|  |  |  |
| **Dual Use Research of Concern (DURC)** | **Indicate where provided: section/submission form** | **N/A** |
| If study is subject to dual use research of concern regulations, state the authority granting approval and reference number for the regulatory approval. |  | X |

**Analysis:**

| **Attrition** | **Indicate where provided: section/figure legend** | **N/A** |
| --- | --- | --- |
| Describe whether exclusion criteria were pre-established. Report if sample or data points were omitted from analysis. If yes, report if this was due to attrition or intentional exclusion and provide justification.  Exclusion criteria were pre-established. Please, see above for more details on the exclusion criteria. The excluded data were then omitted from analysis. This was intentionally done to prevent strong initial preferences from biasing the three-chamber sociability results, as mentioned above. | Materials and methods (*Statistical analysis*) |  |
|  |  |  |
| **Statistics** | **Indicate where provided: section/figure legend** | **N/A** |
| Describe statistical tests used and justify choice of tests.  Within each sex, the three-way repeated measures analysis of variance (ANOVA) with pretreatment (vehicle *vs.* antalarmin) and treatment (saline *vs.* morphine) as between-subjects factors and side (mouse *vs*. object) or test phase (habituation *vs.* sociability) as a within-subject factor was used to analyze time spent in the regions of interest (ROIs) or distance travelled during the three-chamber test by C57BL/6J mice.  A three-way repeated measures ANOVA with genotype (CRF_1_ WT *vs.* CRF_1_ HET *vs.* CRF_1_ KO) and treatment (saline *vs.* morphine) as between-subjects factors and side (mouse *vs*. object) or test phase (habituation *vs.* sociability) as a within-subject factor was used to analyze time spent in the ROIs or distance travelled during the three-chamber test by CRF_1_ receptor-deficient mice.  The two-way ANOVA with pretreatment (vehicle *vs.* antalarmin) or genotype (CRF_1_ WT *vs.* CRF_1_ HET *vs.* CRF_1_ KO) and treatment (saline *vs.* morphine) as between-subjects factors was used to analyze sociability ratio and the firing frequency (Hz) results of the electrophysiology studies.  Sociability ratio and firing frequency displayed by C57BL/6J mice were also examined by a three-way ANOVA with sex (males *vs.* females), pretreatment (vehicle *vs.* antalarmin) and treatment (saline *vs.* morphine) as between-subjects factors.  The accepted value for significance was P<0.05. Following significant interaction effects, the Newman-Keuls post-hoc test was used for individual group comparisons.  The statistical tests mentioned above were appropriate to the experimental designs employed.  The exact values of N are reported in the **Supplementary files 1a-c**. Exact p-values are also reported in the **Supplementary file 1**.  Values in the figures represent mean±SEM. | Materials and methods (*Statistical analysis*) |  |
|  |  |  |
| **Data availability** | **Indicate where provided: section/submission form** | **N/A** |
| For newly created and reused datasets, the manuscript includes a data availability statement that provides details for access (or notes restrictions on access).  Yes, the manuscript includes a data availability statement that provides details for access. | In the manuscript, after the “Materials and methods” section. |  |
| When newly created datasets are publicly available, provide accession number in repository OR DOI and licensing details where available.  All of the data are available as a Dryad dataset titled "Piccin et al. eLife 2025 for Dryad" and can be accessed using the following digital object identifier: <https://doi.org/10.5061/dryad.5hqbzkhgj> or URL: [http://datadryad.org/stash/share/d2X3E5ii1QzqAhinw2eevQ0tNBIgZqiLEbpj2kq63iE](http://h52wvrds.r.us-west-2.awstrack.me/L0/http:%2F%2Fdatadryad.org%2Fstash%2Fshare%2Fd2X3E5ii1QzqAhinw2eevQ0tNBIgZqiLEbpj2kq63iE/1/010101946ee3d229-4b19ca44-b927-473c-8178-0ef8134f18ce-000000/RV6_J2sihWVBPfVTI7_lS7RGEmo=410) | In the manuscript (after the “Materials and methods” section) and in the submission form. |  |
| If reused data is publicly available provide accession number in repository OR DOI, OR URL, OR citation. |  | X |
|  |  |  |
| **Code availability** | **Indicate where provided: section/figure legend** | **N/A** |
| For any computer code/software/mathematical algorithms essential for replicating the main findings of the study, whether newly generated or re-used, the manuscript includes a data availability statement that provides details for access or notes restrictions. |  | X |
| Where newly generated code is publicly available, provide accession number in repository, OR DOI OR URL and licensing details where available. State any restrictions on code availability or accessibility. |  | X |
| If reused code is publicly available provide accession number in repository OR DOI OR URL, OR citation. |  | X |

**Reporting:**

The MDAR framework recommends adoption of discipline-specific guidelines, established and endorsed through community initiatives.

| **Adherence to community standards** | **Indicate where provided: section/figure legend** | **N/A** |
| --- | --- | --- |
| State if relevant guidelines (e.g., ICMJE, MIBBI, ARRIVE, STRANGE) have been followed, and whether a checklist (e.g., CONSORT, PRISMA, ARRIVE) is provided with the manuscript.  ARRIVE guidelines were followed. A checklist was not created and is not provided with the manuscript. | Materials and methods (*Animals*) |  |
